# Optical Biosensors for the Diagnosis of COVID-19 and Other Viruses—A Review

**DOI:** 10.3390/diagnostics13142418

**Published:** 2023-07-20

**Authors:** Pauline John, Nilesh J. Vasa, Azhar Zam

**Affiliations:** 1 Division of Engineering, New York University Abu Dhabi (NYUAD), Abu Dhabi 129188, United Arab Emirates; 2 Department of Engineering Design, Indian Institute of Technology Madras, Chennai 600036, India; njvasa@iitm.ac.in; 3 Tandon School of Engineering, New York University, Brooklyn, NY 11201, USA

**Keywords:** COVID-19 virus, optical spectroscopy, nanomaterials, interferometry, optical biosensors, optical diagnosis, optical therapy, artificial intelligence, Internet of Things, machine learning

## Abstract

The sudden outbreak of the COVID-19 pandemic led to a huge concern globally because of the astounding increase in mortality rates worldwide. The medical imaging computed tomography technique, whole-genome sequencing, and electron microscopy are the methods generally used for the screening and identification of the SARS-CoV-2 virus. The main aim of this review is to emphasize the capabilities of various optical techniques to facilitate not only the timely and effective diagnosis of the virus but also to apply its potential toward therapy in the field of virology. This review paper categorizes the potential optical biosensors into the three main categories, spectroscopic-, nanomaterial-, and interferometry-based approaches, used for detecting various types of viruses, including SARS-CoV-2. Various classifications of spectroscopic techniques such as Raman spectroscopy, near-infrared spectroscopy, and fluorescence spectroscopy are discussed in the first part. The second aspect highlights advances related to nanomaterial-based optical biosensors, while the third part describes various optical interferometric biosensors used for the detection of viruses. The tremendous progress made by lab-on-a-chip technology in conjunction with smartphones for improving the point-of-care and portability features of the optical biosensors is also discussed. Finally, the review discusses the emergence of artificial intelligence and its applications in the field of bio-photonics and medical imaging for the diagnosis of COVID-19. The review concludes by providing insights into the future perspectives of optical techniques in the effective diagnosis of viruses.

## 1. Introduction

### 1.1. History of Viral Pandemic Diseases

In recent times, COVID-19 played havoc, causing escalating mortality rates globally. According to the World Health Organization (WHO), on 21 February 2023, 757,264,511 confirmed cases and 6,850,594 deaths related to COVID-19 were reported globally [1]. The history of viral pandemic diseases, including the Spanish flu, which originated in 1918, severe acute respiratory syndrome (SARS), and the Middle East respiratory syndrome (MERS), which originated a decade ago, and the recent emergence of a novel coronavirus caused by the SARS-CoV-2 virus, is tabulated in Table 1.

### 1.2. The Major Characteristics of Coronavirus

The MERS, SARS, and SARS-CoV-2 viruses are considered the most lethal respiratory diseases transmitted by zoonotic transmission, leading to death in patients with severe comorbid conditions [16,17]. They are highly contagious among humans. Each type of virus is distinctively characterized based on its surface proteins and lipid profiles. Coronavirus belongs to the subfamily Coronaviridae and is an enveloped RNA virus 100–160 nm in diameter, with a spherical structure. This subfamily, based on phylogenetic relationships and genomic structures, is classified into four groups: (i) alpha coronavirus, (ii) beta coronavirus, which infects mammals, (iii) gamma coronavirus, which infects aves, and (iv) delta coronavirus, which infects both aves and mammals [16]. Coronavirus has the single-stranded positive-sense RNA (ssRNA) genetic material and the largest genome of 26.4–31.7 kb, which encodes the structural proteins such as spike glycoprotein (S), membrane glycoprotein (M), nucleocapsid interrupts phosphor protein (N), and envelope (E) protein [16,17,18]. The genome of SARS-CoV-2 shares 82% of its sequence identity with SARS-CoV and MERS-CoV and more than 90% of its sequence identity with that of structural proteins and essential enzymes. Coronavirus uses the spike protein (S) to bind to the receptor of the host cell surface and initiate infection. The mechanism of host entry differs for different coronaviruses [19]. A more detailed discussion on the genomics, proteomics, and mechanism of pathogenesis of SARS-CoV-2 has been reported as a breakthrough therapy [16].

### 1.3. Biological Specimens for SARS-CoV-2 Detection

The spread of acute respiratory viruses between humans occurs through direct or indirect contact with respiratory droplets containing coronavirus RNA, of coarse (more than 5 μm aerodynamic diameter) or fine (less than or equal to 5 μm aerodynamic diameter) aerosols [20]. There are several specimens, including throat swabs, nasal swabs, anal swabs, blood, saliva, sputum, and bronchoalveolar lavage fluid in the lungs, from which SARS-CoV-2 is detected [20,21,22,23]. In addition to the aforementioned specimens, research groups have conducted experiments using an optical particle counter and reverse transcriptase polymerase chain reaction (RT-PCR) to study the presence of the viral RNA of influenza virus, rhinovirus, and coronavirus [24,25]. In the studies conducted to test the effectiveness of wearing masks, it has been confirmed that aerosol transmission from infected subjects without masks is a potential mode of transmission of coronavirus [26]. Hence, exhaled breath could also be considered as a significant specimen for the non-invasive detection of coronavirus.

### 1.4. Conventional Diagnostic Techniques for SARS-CoV-2

**Figure 1:**
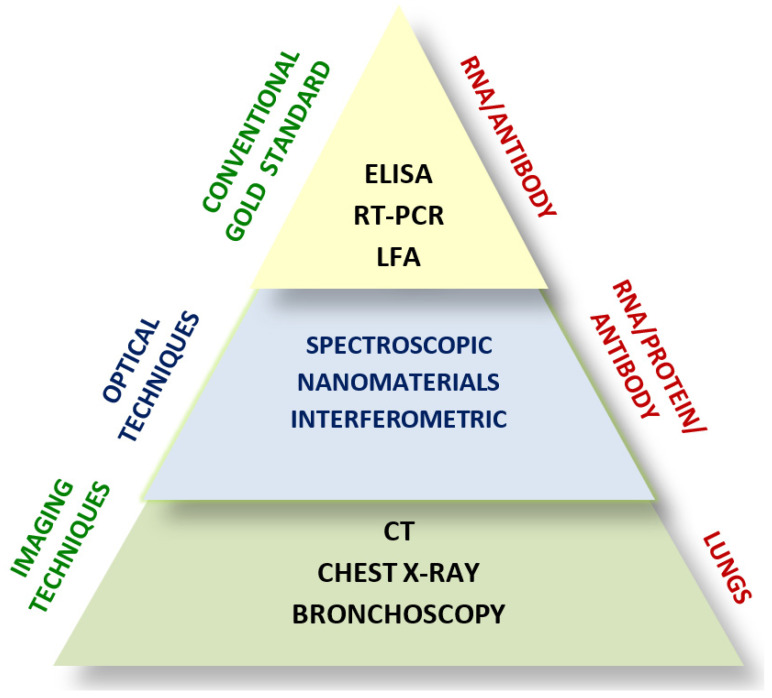
A schematic representation of different diagnostic methods for COVID-19. ELISA—enzyme-linked immunosorbent assay, RT-PCR—Reverse transcriptase polymerase chain reaction, LFA—Lateral flow assay, CT—Computed tomography.

The alarming increase in the number of cases and deaths caused by SARS-CoV-2 has urged the research community to develop innovative diagnostic and therapeutic techniques to combat the impact caused by coronavirus worldwide. Figure 1 shows the schematic of a broad classification of various COVID-19 diagnostic techniques. Conventional methods that provide quantitative analysis, such as enzyme-linked immunosorbent assay (ELISA), polymerase chain reaction (PCR), Western blotting, and immunofluorescent assay, are based on the detection of (i) antibody (immunoglobin Ig M, IgA, and IgG) production, (ii) virus antigen, (iii) protein, and (iv) nucleic acid for viral DNA and RNA [27]. These methods are clinically approved for virus diagnosis because of their robustness and repeatability. Although they are considered gold standards for virus detection, they lack in terms of accuracy, cost-effectiveness, and speed. 

PCR, a well-established diagnostic technique, is considered to possess sensitivity of several orders of magnitude higher than that of serological tests; however, real-time large-scale routine screening is challenging because of the need for expensive equipment, laborious procedures performed by skilled personnel, ease of contamination, and longer duration of data processing and analysis. Besides these limitations, high rates of false-positive and false-negative results are also other drawbacks of the PCR method [28]. Relatively, ELISA technique has a low sensitivity and requires high-quality sample preparation, which limits its use for in situ detection [29].

Qualitative imaging techniques such as computed tomography (CT), chest X-ray, and bronchoscopy are also used in the diagnosis of COVID-19 [29]. The limitations of these methods are their high cost and low resolution. Several reviews have been reported on the improvements in the clinical diagnostic techniques that are currently being used for the diagnosis of COVID-19 [30,31,32]. The development of a non-invasive, rapid, and low-cost diagnostic system based on novel techniques to improve the sensitivity and specificity of virus detection is crucial. The advantages of optical imaging techniques over these conventional imaging techniques are their high resolution, non-ionizing nature, non-invasive attributes, portability, short processing time, and low equipment cost [33].

This review presents in detail the different optical virus detection techniques broadly classified into (i) spectroscopic and nanomaterials-based optical biosensors and (ii) interferometry-based optical biosensors. This review also highlights the importance of point-of-care biosensors, including (iii) lab-on-a-chip-based optical biosensors and (iv) cutting-edge smartphone-incorporated optical biosensors, for providing ease in the detection of viruses. The application of (v) smart optical biosensors based on artificial intelligence for efficient screening, and diagnosis of COVID-19 is also included. In addition to optical diagnostic techniques, this review also discusses the optical therapeutic revenues that could alleviate the problems faced by the rapid spreading of COVID-19.

## 2. Optical Biosensors

### 2.1. Spectroscopy and Nanomaterials-Based Optical Biosensors

Advancements in science and technology have led to the growth of spectroscopic techniques in clinical and biological studies. A retrospective review of spectroscopic techniques used in the diagnosis of viral infection for a decade (2006–2016) has been reported by one of the research groups and includes nuclear magnetic resonance spectroscopy, near-infrared spectroscopy, Raman spectroscopy, surface-enhanced Raman spectroscopy (SERS), and molecular fluorescence spectroscopy [34]. In addition to spectroscopy-based optical techniques such as Raman spectroscopy and SERS, molecular fluorescence spectroscopy and infrared spectroscopy have also been demonstrated for detecting viruses [35,36,37,38,39,40]. The non-optical spectroscopic nuclear magnetic-resonance-based method has also been discussed for the detection of viruses [41,42]. Processing and analyzing large amounts of spectroscopic data is challenging, requiring computational analysis, which includes pre-processing, and multivariate analysis such as principle component analysis (PCA), cluster analysis (CA), genetic algorithm (GA), successive projections algorithm, the partial least square (PLS) method, and the linear regression analysis (LRA) method, mostly combined with PCA and PLS [34].

The immense potential of the spectroscopic technique and the need to utilize the benefits it offers in virology studies is emphasized in this section.

#### 2.1.1. Raman Spectroscopy

Raman spectroscopy works on the phenomenon of inelastic scattering, wherein a monochromatic light source is employed in detecting the presence of polar and non-polar chemical bonds present in the sample, along with their cellular changes. In addition to their non-destructive, reagent-less, non-contact capability of detecting the unique spectral fingerprints of molecules, the insignificant Raman scattering of water is the main advantage of utilizing this approach in biological studies compared to other techniques like NIR spectroscopy and Fourier transform IR spectroscopy.

Raman spectroscopy and surface-enhanced Raman spectroscopy techniques have been widely considered for the detection of several types of viruses such as herpes simplex virus type 1 (HSV-1), hepatitis C virus, ALVAC virus, tobacco mosaic virus, and several other emerging new influenza viruses [35,36,37,38,39,40,43]. The detection of structural changes of nucleic acids, proteins, and lipids of HSV-1 was observed with a sensitivity close to 100% in the Raman spectrum range of 1195–1726 cm^−1^, by which they could differentiate the controlled group from the infected cells [44].

**Figure 2:**
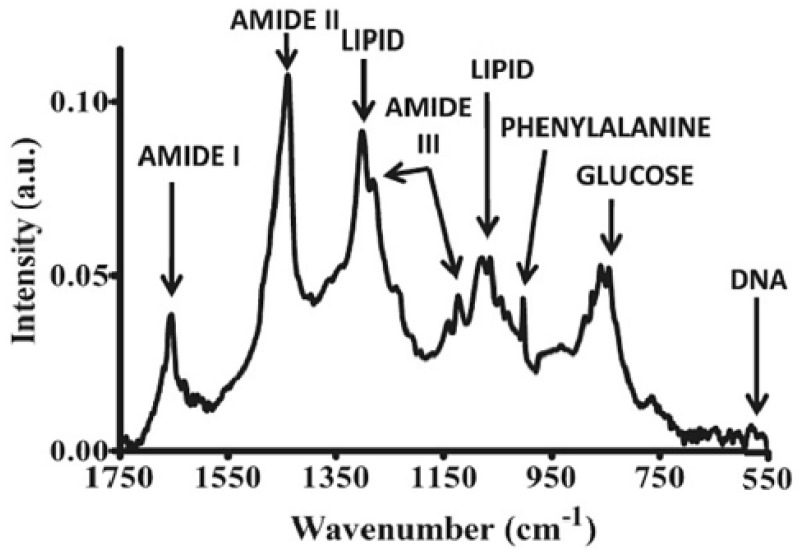
Assignments of Raman peaks for various biological molecules. Reprinted (adapted) with permission from [45]. Copyright 2011 American Chemical Society.

Another group has demonstrated Raman spectroscopy-based label-free early detection of adenovirus-infected human embryonic kidney epithelial (HEK293) cells at 12, 24, and 48 h after instigating the infection [45]. Principle component analysis has been incorporated for classifying infected cells from control cells. A continuous wave Ti:sapphire laser source with an excitation wavelength of 785 nm has been used to detect bands at 1003 cm^−1^ and 1440 cm^−1^, which are attributed to phenylalanine and the CH deformation mode, respectively. As shown in Figure 2, bands at 1655 cm^−1^, 1448 cm^−1^, and 1337 cm^−1^ have been attributed to the amide I, CH bending, and amide III modes of protein, respectively [45]. A band at 1089 cm^−1^ has been observed as a result of the symmetric stretching vibration mode of the phosphate, and at 850 cm^−1^ it has been attributed to DNA [36], whereas a good biomarker for the proliferation of the virus in cells and the defense response of the cells has been observed at 952 cm^−1^ band, attributed to the ${\mathrm{PO}}_{4}^{3-}$ group 48 h after the virus has been introduced [45]. SERS-based techniques in conjunction with multivariate analysis such as PCA and hierarchical cluster analysis (HCA) have been reported elsewhere for detecting respiratory syncytial virus (RSV) [46].

A recent patent contributes to the development of a hand-held micro-Raman portable device for the detection of protein-based compounds such as bacteria, fungi, and viruses, including various influenza viruses (H1N1 and H2N3) [47]. They have claimed to have detected Raman shifts from 700 to 1700 cm^−1^ approximately, with distinct peaks for various viruses. To distinguish various virus strains, analysis of peak ratios and shifts is essential. In this method, an excitation wavelength of 785 nm and narrow spectral bands of 640–740 cm^−1^, 1200–1260 cm^−1^, 1520–1560 cm^−1^, and 1640–1740 cm^−1^ have been considered for detecting distinct carbon–carbon nucleic acids and other amide groups of target pathogens [47].

#### 2.1.2. Near-Infrared and Fourier Transform Infrared Spectroscopy

**Figure 3:**
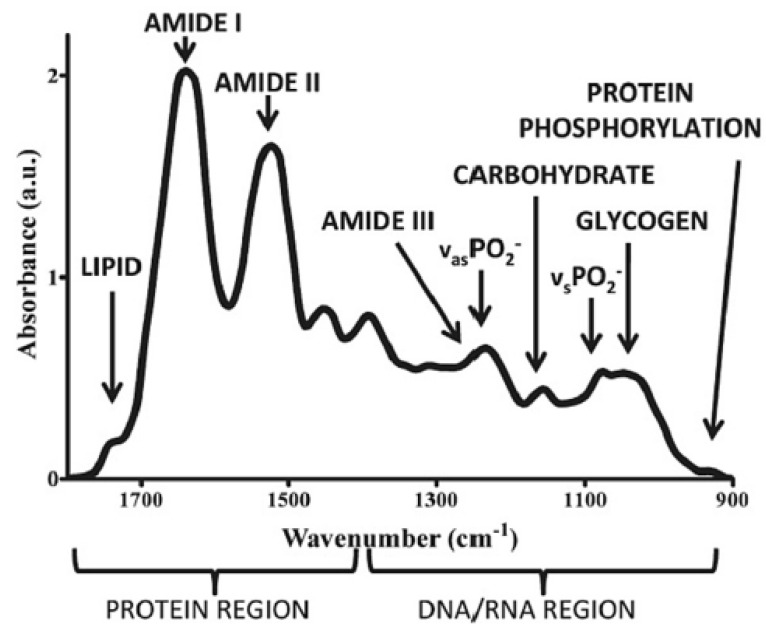
Infrared fingerprints of various biological molecules. Reprinted (adapted) with permission from [45]. Copyright 2011 American Chemical Society.

Non-invasive and nondestructive near-infrared (NIR) spectroscopy has been widely used in several clinical applications, mainly because of the fast detection, without requiring reagents or sample preparation. The “optical window” region of 650–1100 nm in the near-infrared (NIR) wavelength region of 700–2500 nm is the suitable region for the measurement of biomolecules such as proteins, lipids, and carbohydrates [40]. Stretching, bending, rocking, and scissoring are the various vibratory motions of different chemical bonds that result in absorption in the infrared region. The stretching and bending vibrations of functional groups of C-H, O-H, and N-H bonds result in absorption in the NIR region. Detection of human immunodeficiency virus type-1 (HIV-1) subtypes using NIR spectroscopy has been demonstrated [38]. Absorption wavelengths at 682 nm, 948 nm, 1028 nm, and 1058 nm were used to discriminate different subtypes of HIV-1. Absorption around 950 nm has been considered to be the prominent peak for HIV-1, which occurs as a result of the absorption combination tone of (2ν_1_ + ν_2_), i.e., symmetric stretching vibration of O-H (ν_1_) and asymmetric stretching vibration of O-H (ν_2_) [40]. On the other hand, in the mid-IR region of 400–4000 cm^−1^, a range of 900–1800 cm^−1^ is attributed to the “bio fingerprint” region for sensing biological samples. The spectral bands at ~1750 cm^−1^, 1155 cm^−1^, 1650 cm^−1^, 1550 cm^−1^, 1260 cm^−1^, 1225 cm^−1^, and 1080 cm^−1^ have been observed to be attributed to lipids, carbohydrates, proteins amide I, amide II, amide III, DNA, and RNA, respectively, as shown in Figure 3 [45].

Comparison of the NIR-based Raman spectroscopy technique with standard serological ELISA test and the molecular PCR technique has been demonstrated for classifying healthy human blood serum and viral hepatitis-C-infected human blood serum [48]. This method utilizes a semiconductor laser source with an excitation wavelength at 830 nm and incorporates multivariate analysis methods such as principal component analysis (PCA) for spectral feature extraction and the Mahalanobis distance method for blood sample classification. The Raman bands at 1170 cm^−1^, 1257 cm^−1^, and 1344 cm^−1^ are attributed to the CO-O-C asymmetric stretching in lipids and CH_2_ wagging band in biomarkers—phospholipids get activated by the hepatocytes process in hepatitis C blood serum. These significant bands at 1002 cm^−1^, 1170 cm^−1^, 1257 cm^−1^, and 1344 cm^−1^ are the regions where the most prominent differences between healthy and hepatitis C spectra were reported [48]. The NIR Raman-spectroscopy-technique-based detection, compared to conventional chemiluminescence analysis, was reported to classify hepatitis-C-infected human blood serum from healthy human blood serum with a sensitivity of 92%. 

Attenuated total reflection–Fourier transform infrared (ATR-FTIR) spectroscopy based on the absorption or transmission of the evanescent wave by the sample is advantageous in the detection of viruses, as it does not require reagents or sample preparation, provides good spatial resolution, and is a nondestructive method. However, the spectral overlap between cells and viruses in the region between 1800 and 900 cm^−1^ is one of the disadvantages [49]. The application of ATR-FTIR spectroscopy integrated with multivariate approaches used in the detection of changes in biological samples caused by viruses has been elaborated [50]. A list of various optical spectroscopic techniques, target viruses, the specific spectral range used for detection, and corresponding sensitivity achieved for each technique is included in Table 2.

**Table 2:** List of various optical spectroscopic techniques, the spectral range used for detection of the corresponding target viruses, and the sensitivity achieved for each technique.

Spectroscopic Techniques	Wavelength/Wavenumber	Target Virus	Sensitivity	Ref(s)
Raman	800–1700 cm^−1^	Adenovirus	-	[36]
Raman and FTIR	750–1600 cm^−1^ and 1500–1800 cm^−1^	Hepatitis C virus	-	[38]
Raman	1195–1726 cm^−1^	Herpes simplex virus type 1	100%	[44]
SERS	600 cm^−1^ to 4500 cm^−1^	SARS-CoV-2	97%	[51]
Raman	500 to 3800 cm^−1^	RNA virus	92.5%	[52]
NIR Raman	1002, 1169, 1262, and 1348 cm^−1^	Hepatitis C virus	92%	[48]
NIR	950, 1030, and 1060 nm	Human immunodeficiency virus-1	-	[40]
ATR-FTIR	1800 to 900 cm^−1^	Dengue virus	100%	[49]
ATR-FTIR	4000−650 cm^−1^	SARS-CoV-2	95%	[53]

#### 2.1.3. Gold-Nanoparticle-Based Surface Plasmon Resonance

The optical, mechanical, electronic, and magnetic properties of nanomaterials, such as metal nanoparticles, carbon nanotubes, silica nanoparticles, fluorescent quantum dots, plasmonic gold nanoparticles, silver nanocrystals, and polymeric nanoparticles, have a great impact on clinical research and have envisioned applications in biosensing, biomedical imaging, and clinical diagnosis [54]. Noble nanoparticles, specifically metal and gold nanoparticles (AuNPs) ranging from 1 to 800 nm in size, used as nanoprobes for the diagnosis of various types of viruses have outpaced other materials because of their outstanding stability, biocompatibility, strong fluorescence, excellent photostability, and long emissive lifetimes and their intense color providing ease in visualizing as labeling agents. They have been inevitable in detecting the targets of pathogenic viruses, such as the antigens, capsid proteins, and specific gene segments in their genome. They are also observed to form stable bioconjugates with biomolecules to aid in virus detection with high sensitivity and specificity [55]. They are synthesized using several methods such as ultraviolet irradiation, laser ablation, lithography, and photochemical reduction of Au to form different morphological shapes and hollow structures. Resonance light scattering detection using localized surface plasmon resonance (LSPR) and Raman spectroscopy, color amplification detection using the colorimetric technique, and fluorescence quenching, and fluorescence enhancement are the optical signal transduction functions of AuNPs in the detection of viruses [56,57,58,59,60,61,62,63]. LSPR-based detection of influenza virus particles by conjugating a peptide linker with a QD and AuNP, demonstrating alteration in the intensity of absorption in the presence of influenza virus particles, has been reported [64]. A recent review reports in detail the outstanding capability of AuNPs in detecting several human virus groups, including the group Coronaviridae [65]. Intense surface plasmon resonance (SPR) bands of AuNPs exist between 510 and 1100 nm. SPR-based biosensors for the detection of the dengue virus in its early stage has been reported [65]. The size of the entire optical SPR biosensor and the regulation of temperature are the challenges reported. Based on the optical characteristics, such as phase, intensity, angular, wavelength, and polarization modulation, SPR-based biosensors have a wide application in biosensing, immune sensing, detection of blood protein, refractive index measurements, polarization fibers, spectrometers, and microscopy [66,67,68,69,70].

Experimental and computational investigation of the binding reaction between the analyte (antibodies, Ab) and the ligand (antigens, Ag) (dengue virus serotype 2) using angular modulation of SPR biosensor has been reported [71]. The ligand or the reactant antigen is immobilized on the gold surface (~50 nm) coated on the outer surface of the prism (fused silica, with a refractive index of 1.457), while the analyte is allowed to flow on the gold surface. A 100 nm thick carboxymethyl–dextran matrix is used as a linker layer to provide an inert hydrophilic environment for efficient immobilization. The interaction of the antigens and antibodies leads to binding, which in turn leads to changes in the refractive index in the vicinity of the immobilized surface antigens. A light beam of wavelength 633 nm strikes the inner surface of the prism at an angle where it is totally reflected after generating an evanescent wave that propagates through the binding medium and interacts with the mobile electrons in the gold film. At this incident angle, the plasmon resonance generated by the excited electrons is detected from the reflected light beam with reduced intensity. According to this study, the change in the refractive index of binding medium and serum, for low-, mid-, and high-positive patients were 6.91 × 10^−3^, 9.095 × 10^−3^, and 10.532 × 10^−3^, resulting in the SPR angle variation of 0.6910°, 0.9095°, and 1.0532°, respectively [71]. Despite the practicality of SPR in the detection of viruses, high fabrication cost, the requirement of high excitation power, and toxicity, except for gold, are the major challenges of SPR detection [33].

#### 2.1.4. Gold-Nanoparticle-Based Surface-Enhanced Raman Spectroscopy

The surface-enhanced Raman spectroscopy (SERS) approach is a substitute to overcome the limitation of detecting weak signals generated in conventional Raman spectroscopy and is said to have sensitivity and specificity from 10^4^ to 10^9^ higher than conventional Raman spectroscopy. It is a widely used application in detecting viruses, cancerous cells, and biological imaging. An indirect method of detecting specific viruses by tagging the antibodies induced by them with nanoparticles of 150 nm has been demonstrated by generating surface-enhanced Raman spectroscopy signals [72]. The SERS active Ag-Au nano-wave chip, functionalized by a DNA probe, has also been employed in the detection of specific oligonucleotide sequences of the dengue virus with fewer false-positive errors [73]. A comprehensive review including various biosensors working on optical, electrochemical, and microfluidic approaches has been elaborately discussed for the detection of biomarkers of the dengue virus [74].

The gold nano-star-based SERS technique has also been demonstrated as a nanoprobe for the identification and quantification of RNA mutations in the influenza A virus genome, and vibrational modes at 1590 cm^−1^, 1468 cm^−1^, 1391 cm^−1^, and 1196 cm^−1^ were observed [75]. This technique has been reported to offer high sensitivity and has been a favorable technique for single-cell-level target detection. Another group has demonstrated AuNP-SERS-based detection of different types of influenza viruses (100 nm in diameter) using a CW laser source with an excitation wavelength at 785 nm, and distinct Raman peaks have been measured at 923 cm^−1^ and 1356 cm^−1^, for a newly emerged influenza virus strain, and Raman peaks at 740 cm^−1^ and 1107 cm^−1^, for a lab-adapted influenza virus strain [76]. These differences in Raman peaks for different viruses have resulted depending on the interaction of surface proteins of the virus particles with AuNPs (SERS influenza). The tremendous potential of gold nano-star-encoded SERS conjugated with antibodies has also been reported for detecting viral biomarkers for Zika and dengue virus, with detection limits of 0.72 ng mL^−1^ and 7.67 ng mL^−1^, respectively [77]. It has been suggested that the early detection capability of SERS could have a great impact on biosensing combined with lateral flow assays for point-of-care diagnostic applications in the future [78].

Recently, another group has demonstrated SERS-coupled multivariate analysis for ultra-fast detection of SARS-CoV-2, without the need for RNA extraction. In this method, strong SERS signals were obtained at 1032 cm^−1^ (phenylalanine), 1051 cm^−1^ (C-N stretching in protein), 1089 cm^−1^, 1189 cm^−1^ (Amide III for C-N stretching and N-H bending), 1447 cm^−1^ (CH_2_ bending mode of proteins), and 1527 cm^−1^ for silver nanorod SERS array functionalized with human cellular receptor angiotensin-converting enzyme 2 (ACE2), using a near-infrared (NIR) confocal microscope equipped with a 785 nm NIR laser source [79]. The spectral intensity of most of the peaks was quenched and a red shift from 1189 to 1182 cm^−1^ (N-H bending in response to H-bond of ACE2) was observed as a result of the receptor-binding domain of subunit S1 of SARS-CoV-2 spike proteins, recognition, and binding on SERS array. These studies were conducted in 17 water samples collected from hospitals and pipe networks in Wuhan, China. An accuracy of 93.33% for the ratio 1189/1182 with a false-positive and false-negative percentage of 10% and 0%, respectively, were estimated, and the 1189/1182 ratio has been a satisfactory biomarker for the diagnosis of SARS-CoV-2 presence in water samples [79]. An ultrafast, portable diagnostic screening tool based on this approach has been suggested for public health.

#### 2.1.5. Magnetic-Nanoparticle-Based Fluorescence Biosensors

Quantum dots used in fluorescence biosensors are advantageous in terms of high quantum yield, tunability of emission wavelength, photostability, and Stokes shift compared to small-molecule organic dyes. However, their high cytotoxicity in oxidative environments and the possible damage of DNA is a major concern for long-term in vivo studies [33]. As an alternative, conjugated polymer nanoparticles and carbon dots are developed as biocompatible light-emitting nanomaterials. Optical detection of coronavirus by synthesizing chiral zirconium quantum dots (Zr QDs) of 2–3 nm size was reported to exhibit fluorescence at 412 nm and absorbance at 378 nm [80]. A change in the fluorescence intensity with varying concentrations of virus solution has been reported. Biosensing of coronavirus and infectious bronchitis virus (IBV) with a detection limit of 79.15 EID/50 μL has been reported by the fluorescence properties of nanohybrid conjugate with quantum dots and magneto-plasmonic nanoparticles, through separation by an external magnetic field. It is also reported that this technique has achieved 10 times higher sensitivity compared to the conventional ELISA technique [80]. The promising features of Zr QDs, such as optical chirality, nontoxicity, biocompatibility, strong fluorescence emission tunable across the visible and infrared ranges, broad excitation wavelength, optical and thermal stability, mechanical strength, and better quantum yield, would significantly benefit their use in the field of biosensing [80]. The tremendous potential of these nanoparticle-based biosensors could replace or enhance the performance of existing virus detection methods. Optical fluorescence-based biosensors are widely used in the detection of viruses because of their accuracy and sensitivity in detection. A three-dimensional copper-based metal–organic framework has been utilized based on the fluorescence technique to detect dengue and Zika viruses with detection limits of 184 and 121 pM, respectively [81]. Table 3 provides a list of nanomaterial-based optical techniques, their corresponding target viruses, and detection limits.

The successful applications of optical biosensor technologies used for the detection of viruses listed in Table 2 and Table 3 assure the capability of these optical techniques to be used for detecting SARS-CoV-2 as well, with an enhanced specificity. Despite the potential of nano-biosensors for the detection of viruses, there are certain challenges such as precise manipulation of the size of nanoparticles, low phototoxicity, low background fluorescence, low photodamage, high photostability of probes, reproducibility, and high throughput that need to be addressed [89].

### 2.2. Interferometry-Based Optical Biosensors

The refractive index is one of the fundamental optical parameters used for the label-free sorting of materials, including viruses. It is challenging to detect the refractive index of biological material with high precision because of its heterogeneous nature. Application of optical cavity resonance, surface plasmon resonance, and optical interferometry approaches aid in indirect measurement of the refractive index by the measurement of spectral shift, size of the virions, and density of the virions bound on an antibody-sensing surface [90]. This phenomenon of refractive-index-based spectral shift, which is attributed to the change in the concentration of the analyte such as glucose, has also been reported for non-invasive and selective detection of the analyte using a frequency domain dual-wavelength low-coherence interferometry system in the NIR wavelength region [91,92].

Image processing of coronavirus images obtained using two-beam and multiple-beam interferometric techniques has also been reported [93]. The contrast of fringe shift obtained in multiple beam interference is claimed to be higher compared to two-beam interference. Furthermore, accurate detection of the virus cell diameter based on the polychromatic spectral distribution of illuminating light related to the refractive index has been suggested. The different types of interferometric system configurations applicable for virus detection are discussed below.

#### 2.2.1. Photonic Crystal Biosensors

Optical label-free photonic crystal biosensors have also been designed and developed for rapid detection of viruses such as the dengue virus, with a high sensitivity, based on the measurement of refractive index change on a photonic crystal [94]. A unique geometry of a photonic crystal biosensor waveguide with five resonators has been designed to distinguish multiple analytes simultaneously. Replacement of bulky spectrometers with vertical-cavity surface-emitting laser systems have also been considered and utilized for detecting human anti-dengue IgG antibodies [95]. However, false-positive errors and low specificity were the drawbacks of this technique.

#### 2.2.2. Back Focal Plane Interferometry

Optical trapping of a single virion with optical tweezers is considered for the measurement of the refractive index and size of the virions, based on their dependence on the stiffness of the optical trap. A novel method based on an optical tweezer using back focal plane interferometry, for measurement of the refractive index of a single human immunodeficiency virus type-1 (HIV-1) with high precision in aqueous media, has been demonstrated using a tapered amplifier diode laser of wavelength 830 nm and a laser power of 130.8 mW, resulting in a refractive index of 1.42, with less than a 2% coefficient of variation [96].

#### 2.2.3. Mach–Zehnder Interferometry

**Figure 4:**
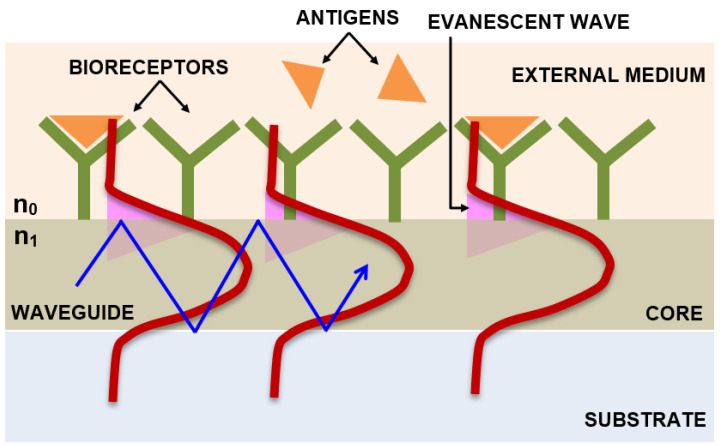
Schematic representation showing the interaction of biomolecules with the waveguide surface within the evanescent field.

Figure 4 describes the mechanism of the evanescent wave, where a variation in the refractive index is detected when there is a biomolecular interaction at the surface of the waveguide. A simple Mach–Zehnder interferometer configuration incorporated with a closed-loop flow system based on the principle of the evanescent wave, which enables measurement of the interference of two light beams (laser diode light source of 670 nm wavelength and 5 mW power), one that passes through a waveguide that acts as a biosensor with antibody coupled on to its surface and the other one that is the reference beam that is not functionalized with the bioreceptor, thereby to detect the avian influenza virus, has been designed. As the target binds to the bioreceptor immobilized on the surface of the waveguide, the water molecules get displaced and its structure gets altered, resulting in the velocity change of the light beam that propagates. The phase of the interference pattern is measured using the Fourier transform algorithm, with a detection sensitivity of 0.01 rad and a change in the refractive index of less than 10^−6^. A detection limit as low as 0.0005 HA (hemagglutination—antigen-specific) units/mL of virus concentrations has been reported [97].

#### 2.2.4. Integrated Optical Young’s Interferometry

**Figure 5:**
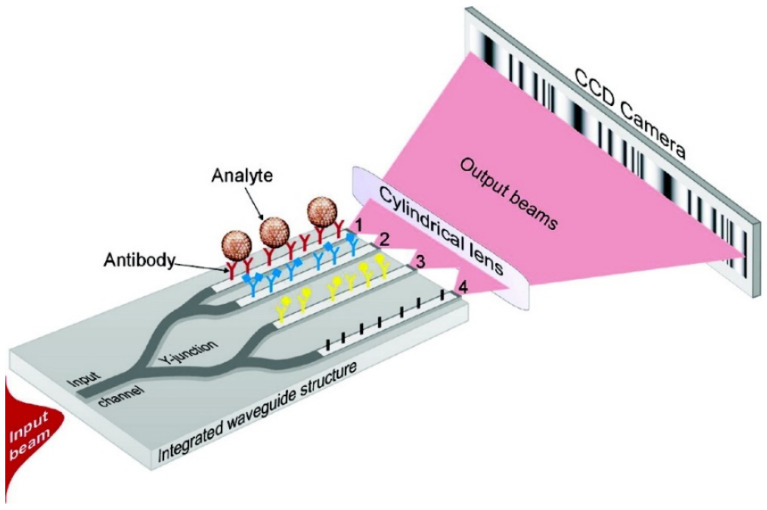
Schematic representation of a four-channel integrated optical Young interferometry sensor. Reprinted (adapted) with permission from [98]. Copyright 2007 American Chemical Society.

A four-channel, integrated optical Young interferometry technique, using a monochromatic argon laser light source and a CCD camera as shown in Figure 5, has been reported for selective and sensitive detection of herpes simplex virus type 1 (HSV-1) at very low concentrations of 850 particles/mL [98]. Three appropriate antibody-coated waveguide channels have been used to measure different analytes, and the fourth channel has been used as a reference channel. A corresponding phase change resulting from the interference pattern measured from the evanescent wave that has been used to probe the analyte binding to the antibody surface has been measured.

For the HSV-1 particle size of 150–200 nm and refractive index of ~1.4, a phase change of ~1.1 × 10^−4^ fringes for a single virus particle has been reported. This system was further developed into a handheld device by integrating a glass microfluidic system with a four-channel Young interferometer optical chip [99]. The goal of this approach was to achieve a shorter response time of 5 s using a disposable chip, compared to the 100 s that was previously achieved using a bulky cuvette, as well as to aid in simultaneous and multiplexed detection of numerous pathogenic species. Another advantage of this system has been the requirement of a smaller quantity of samples in the order of a few microliters. This technique has been suggested further for screening purposes in clinics, airports, and public places to control pandemic outbreaks such as SARS and avian influenza [99].

Another study using Young’s interferometer waveguide has been demonstrated with multiple wavelengths at 400 nm, 500 nm, and 700 nm for the detection of refractive index changes based on different distances from the sensor surface, to distinguish 50–200 nm size viruses (specific binding to the antibody-coated waveguide surface) from 1–10 nm size proteins (non-specific binding to the antibody-coated waveguide surface), thereby eliminating bulk refractive index changes. A minimum detectable limit of virus mass coverage of 4 × 10^2^ fg/mm^2^ has been reported for an assumed phase precision of ≈10^−4^ fringes. In terms of sensitivity, speed, multi-sensing, and point-of-care sensing for virus detection, Young’s interferometry has been outperforming the conventional PCR technique [100]. Although Mach–Zehnder’s and Young’s interferometers are capable of yielding detection limits down to 10^−7^, they still require a long interaction length for the detection of change in the refractive index. Alternatively, resonating photonic structures based on microring, Fabry–Perot, photonic crystal, and whispering gallery mode resonators overcome this limitation with their high-quality factor and detection limit down to 10 pM [101].

#### 2.2.5. Interferometry Reflectance Imaging Sensor

**Figure 6:**
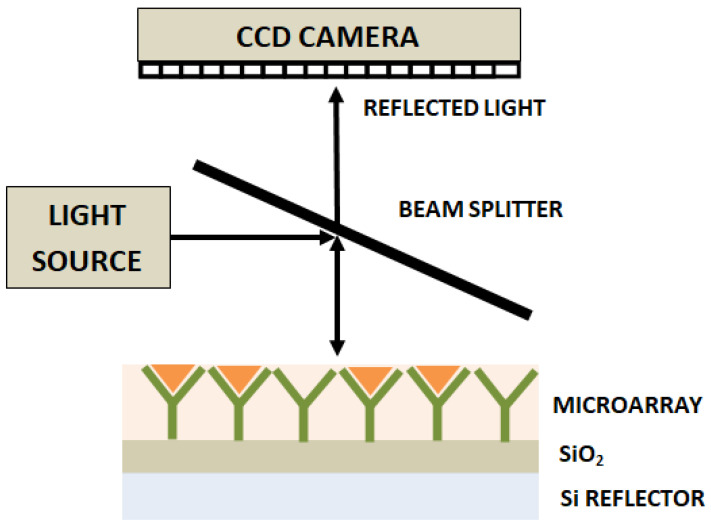
Schematic representation of interferometric reflectance imaging sensor (IRIS).

Another label-free high-throughput technique demonstrated for single-virus and viral antigen detection is the interferometric reflectance imaging sensor (IRIS) [102]. This system is based on an imaging approach that includes measurement of phase changes in the interference response resulting from reflections from a layered substrate, as shown in Figure 6. This approach has been reported to have detected vesicular stomatitis virus, with a detection limit of 5 × 10^3^ PFU/mL.

#### 2.2.6. Hartman Interferometer

In contrast to the Mach–Zehnder and Young’s interferometry techniques, the Hartman interferometer utilizes a planar waveguide and allows interaction of a broad beam of linearly polarized light with the multiple sensing regions on the chip or a waveguide film, fabricated using photolithographic techniques. Individual interferometers have been created on a single chip by immobilizing both specific and nonspecific probes on different regions, thereby allowing multiplexed detection. A photodiode array has been utilized to measure the exiting light from the waveguide. This sensor has been reported to perform the detection of the influenza-A virus with a sensitivity of 2 × 10^6^ PFU/mL [103].

#### 2.2.7. Liquid Core Optical Ring Resonator

A liquid core optical ring resonator (LCORR) has been designed using a cylindrical capillary tube that supports the whispering gallery mode (WGM) [104]. The setup includes an on-chip waveguide-coupled LCORR where the light is coupled through this capillary tube that acts as an optical ring resonator. The analytes are detected as a result of the interaction of the evanescent field from the WGM with the analytes bound to the inner surface of the capillary [105]. Utilizing this technique, the detection of filamentous viruses of 10 nm diameter, with a spectral shift and detection limit of 2 pm and 2.3 × 10^3^ PFU/mL, respectively, has been reported [106]. Although resonant cavity techniques detect biomolecules and viruses with enhanced sensitivity, detection of targets in an abundant background of nanoparticles and the fabrication of resonant-cavity devices into multiplexed configurations to facilitate the detection of different targets in a sample are challenging. Table 4 provides a list of optical interferometric techniques and the corresponding target virus types and the detection limit achieved.

### 2.3. Lab-on-a-Chip-Based Optical Biosensors

Several reviews highlight the need for developing a lab-on-a-chip (LOC)-based technique to replace the time-consuming cell culturing method and expensive electron-microscopy-based identification of viral particles, which necessitates technical skills and expertise. Although flow cytometry, enzyme-linked immunosorbent assay (ELISA) and polymerase chain reaction (PCR) provide high sensitivity and specificity in detecting viruses, they are expensive, time consuming, and labor intensive and require well-trained operators. Although it is possible to couple spectrophotometers, lasers, and microscopes to LOCs, the miniaturization and portability of the detection system is challenging [115]. The introduction of integrated microfluidic LOC devices has an enormous impact on biosensing with a miniaturized platform, initiating cost-effective and rapid diagnosis of viral bodies. Fabrication techniques such as laser ablation, micro-electromechanical system (MEMS) technique, and soft lithography have been used for developing LOC structures [116]. Biodegradable, disposable, and portable paper-based LOC structures such as lateral flow strips (LFS) have also been widely used in diagnostics [116].

An integrated hand-held fluorescent probe system utilizing an LED source to excite the fluorophore at 490 nm has been used to detect the RNA of H5N1 avian influenza virus and Ebolavirus [117]. A similar portable microfluidic PCR platform based on real-time fluorescence detection of hepatitis B virus has been demonstrated with an LED source for exciting the fluorescent dye at a wavelength of 475 ± 10 nm, incorporated with an emission filter at 525 ± 25 nm and a proportional integral derivative algorithm for temperature control. A detection limit of 100 copies/μL DNA was reported to be detected within an hour, with an efficiency of 98.76% [118]. Microfluidic paper-based analytical devices have also been reported to detect the viral protein of 10 ng mL^−1^ in blood and plasma in ≈7 min [119]. A review of different types of nanomaterials, such as zero-, one-, two-, and three-dimensional, used for optical-based POC diagnostics has been presented by another group [120]. An alternative, simple, and highly sensitive test to ELISA has been demonstrated using a graphene-oxide-coated nano-paper on which a suspension of antibodies attached to quantum dots was introduced. The addition of analytes such as bacteria or protein to the nano-paper creates a gap between the quantum dots and graphene oxide, causing fluorescence. This method is considered advantageous compared to the ELISA test, as it is a faster and more portable approach, without the requirement of washing steps.

### 2.4. Smartphone-Based Portable Optical Biosensors

Compact and portable handheld, easily operable point-of-care devices for real-time virus detection are essential in remote locations that lack sophisticated laboratory facilities. Disposable chip sensors based on electrochemical, optical, magnetic, mechanical, thermometric, and microgravimetric quartz crystal microbalance (QCM) methods used for medical diagnostics, food, and environmental analysis have been reported [121]. Among these, optical-technique-based disposable sensors integrated with smartphones is advantageous because of their reliability, sensitivity, non-destructivity, fast sensing, and multiplexing capability [120]. The smart detecting capability of photo cameras, the smart recording capability of image sensors, the smart readout capability of smartphones, and built-in LED that act as smart light sources have led to growing interest among researchers in combining microfluidic chips and micro-biosensors to smartphones for detecting biological constituents such as enzymes, nucleic acids, cells, antigens–antibodies, whole viruses, and microorganisms [122].

A hand-held, fluorescence-based iPhone 5S smartphone used for the in situ detection of proteins with a detection limit of 10 pg/mL has been reported [122]. In this technique, a fluorescence nanoparticle immunoagglutination assay incorporated into an organ-on-chip (OOC)-based smartphone biosensor has been fabricated to enable simulation of the response of human kidneys to nephrotic drugs. Elsewhere, the Y-channel OOC device has been fabricated, wherein the smartphone-based fluorescent microscope consisting of three white LEDs, an objective lens, 480 ± 10 nm bandpass filter for the light source, 500 nm long pass filter for the smartphone camera, 3 V button batteries to power the LED sources, a secondary objective lens, and the smartphone camera, which acts as an image-capturing device are incorporated to measure the fluorescence scatter intensities from the Y-channel OOC. Detection of hepatitis B and human immunodeficiency virus (HIV) with a detection limit of 10 ng/mL using a reflective phantom interface biosensor with an HTC Desire HD smartphone has also been reported [123]. This technique has been suggested to be used for the detection of the presence of multiple targets using monoclonal antibodies and the use of other biomarkers such as proteins and enzymes other than nephrotic drugs. Despite the disadvantages of being a non-label-free approach and causing photobleaching and phototoxicity to mammalian cells, nevertheless, fluorescence imaging has been used for OOC platforms.

Another group has reported the detection of immunoglobin IgG, with a detection limit of 4.25 nmol/L using a smartphone-based photonic crystal biosensor [124]. This biosensor consists of a broadband tungsten halogen light source from which the light beam passes through a linear polarizer, allowing the polarized light to pass through a photonic crystal (PC) surface upon which the protein polymer polyphelysine (PPL) is adhered. The PC design is a one-dimensional grating surface structure, with a grating period of 360 nm and grating depth of 60 nm, on which SiO_2_ of 200 nm thickness and TiO_2_ of 60 nm thickness and a refractive index of 2.35 have been overcoated. This PC acts as a high-efficiency narrow-band reflectance filter, with a center wavelength of 565 nm and a resonant full-width half-max (FWHM) of ~5 nm. A shift in the resonant wavelength has been reported as a result of the adsorption of biomolecules on the PC surface. An accuracy of 0.009 nm shift has been reported [124]. A software application has been implemented for pixel-to-wavelength mapping to reconstruct the resonant wavelength shift from the images captured using the camera of a smartphone that is fixed along with the optical components in the setup.

**Figure 7:**
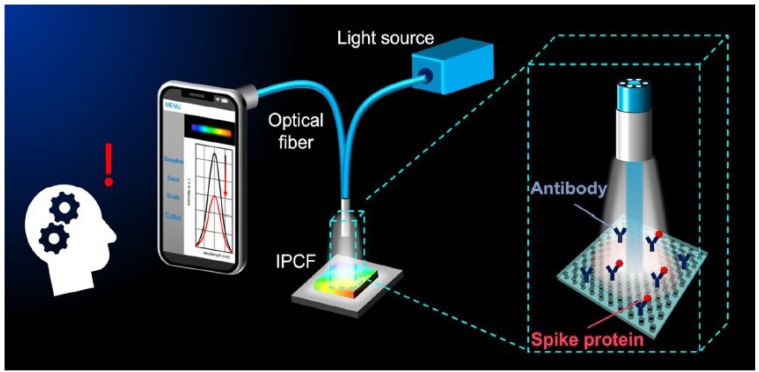
Illustration of a smartphone-based imprinted photonic crystal film (IPCF) sensor designed for optical detection of SARS-CoV-2 spike proteins [125].

**Figure 8:**
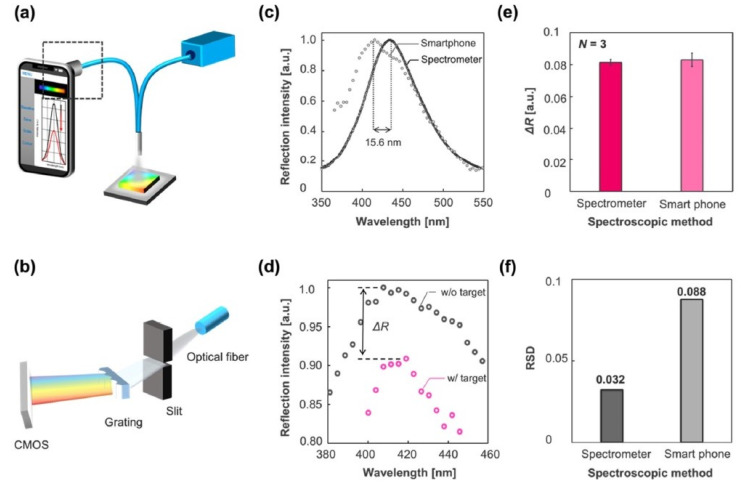
(**a**) Schematic of the optical system incorporated with a smartphone with the square dashed line indicating the smartphone-based spectrometry composed of (**b**) a slit, grating, and CMOS sensor of iPhone 11. (**c**) The reflection spectrum obtained from IPCF using a spectrometer-based system denoted with a black circle and a smartphone-based system denoted using a gray circle, showing a difference in the peak wavelength of 15.6 nm. (**d**) Reflection spectra of the IPCF sensor without the target, denoted with a gray circle, and with the target (after incubation in spike-protein containing 1 ng/mL A. saliva sample solution), denoted with a pink circle. (**e**) Response to the spike protein measured using a spectrometer-based system denoted with a magenta bar and smartphone-based system denoted with a pink bar (N = 3). (**f**) Relative standard deviation (RSD) of the response shown in (**e**), denoting spectrometer result with a black bar and smartphone result with gray bar [125].

Figure 7 shows the schematic of the optical setup developed by incorporating an imprinted photonic crystal film (IPCF) for a reflectometric label-free detection of SARS-CoV-2 spike protein in artificial saliva, with a detection limit of 429 fg/mL. This method uses Go spectro operation software that enables monitoring of the sample in real time and permits the quick display and transfer of data to the cloud. The sensitivity obtained using this method is comparable to a commercial spectrometer-based system, as shown in Figure 8a–f, and is also considered to be cost effective [125]. A detailed review of the progress of a wide variety of smartphones integrated with spectrometry, as well as imaging-based optical biosensors, working on optical theories such as absorbance, fluorescence, reflectance, surface plasmon resonance, and localized surface plasmon resonance used for point-of-care testing, has been reported [126]. The complexity and analytical performance of optical biosensors for implementing smartphone-integrated platforms has been discussed in the review. Although several advantages of these optical biosensors have been discussed, the challenges of smartphone-based optical biosensors include the detection of the analyte without the interference of ambient conditions such as ambient temperature and light, incorporation of simple operations without the need for skilled professionals, compatibility of the hardware of smartphones, and the operation of these optical biosensors with all smartphones, which are the major concerns.

Internet of Things (IoT)-assisted smartphone-based optical biosensors, working on artificial intelligence (AI) platforms, could aid in the sensing or imaging, acquisition, and collection of big data from the sensor, thereby providing support in the prediction of a pandemic outbreak of COVID-19 to track its spread and provide diagnosis and vaccine discovery. With the advances in wearable techniques, wearable smart watches, or wearable patch-integrated smartphones for signal transmission could be invented for rapid and effective screening and tracking of coronavirus-infected subjects, thereby providing efficient control over the spread of the disease [127].

### 2.5. Artificial-Intelligence-Based Smart Optical Biosensors

In the case of imaging techniques, automated detection and classification of large data images of subjects infected with coronavirus are crucial. The emergence of IoT and AI find their applications in tackling big data outcomes from various fields, among which the handling of big data related to health care is greatly beneficial [128,129,130]. Using deep learning (DL) approaches, screening models to differentiate pulmonary CT images of COVID-19 cases from healthy and influenza-A viral pneumonia have been demonstrated with an accuracy of 86.7% [131]. A flow chart with five layers of the AI-based approach in diagnosis and tracing of COVID-19, consisting of (i) input database layer, (ii) selection layer, (iii) imaging layer which includes magnetic resonance imaging (MRI), X-ray, computed tomography (CT), positron emission tomography (PET) and optical microscopy imaging, (iv) optimization layer, and (v) output diagnosis layer, has been described [132]. The traditional optical microscope approach is the main tool used in the investigation of pathological conditions.

A detailed explanation of the various AI-based DL approaches, such as extreme learning machine (ELM), recurrent neural network (RNN), generative adversarial networks (GANs), and long/short-term memory (LSTM), have also been provided for combating COVID-19 [132]. Another group has presented a novel automated screening technique to detect COVID-19 by converting histograms of bio-optical attributes obtained from digital holographic-microscopy-reconstructed red blood cells to feature vectors using the bag-of-features (BoF) method, followed by classification using the linear support vector machine (SVM), with an accuracy, sensitivity, and specificity of 91.67%, 90%, and 92.86%, respectively [133]. The risk of deterioration of patients infected with COVID-19 was predicted automatically by another research group from chest X-rays using a deep convolution neural network along with other clinical predictors, which include vital signs and lab tests. This helps in decision making and prioritizing patients who need immediate emergency treatment [134]. This multi-modal system based on the globally aware multiple instance classifier and gradient boosting model has been successfully used during the first wave of the pandemic at New York University Langone Health and achieved the area under the curve of 0.786. Elsewhere, an IoT-based unmanned aerial vehicle (UAV) incorporated with GPS and thermal cameras to measure human body temperature and a deep learning model to detect people with and without face masks has been proposed to control the spread of the virus [135].

On the other hand, biosensing devices using smart nano-enabled optical and electrochemical biosensors interfaced with AI techniques have been used for rapid early stage diagnosis, point-of-care (POC) application, and management of pandemics efficiently [136]. Even though RT-PCR testing is the primary method of detecting COVID-19, because of the rise in different variants that decreases the sensitivity of testing, a reagentless, label-free, and non-invasive screening approach based on Raman spectroscopy has been demonstrated to detect COVID-19 based on the molecular changes in dried saliva, instead of blood samples. This approach utilized a multiple instance learning-based machine learning (ML) concept and attained an area under the curve of 0.8, with a sensitivity and specificity of 79% and 75%, in males and 84% and 64%, in females, respectively [137]. However, to overcome challenges, such as longer duration of imaging and difficulty in capturing the complete molecular profile of the sample drop, a single-point Raman spectroscopy approach for imaging the whole droplet in a few seconds has been suggested [138]. A comprehensive review proposes the use of nonlinear optics processes that include second-harmonic generation (SHG), sum frequency generation (SFG), nonlinear optical absorption, optical Kerr effect, self-phase modulation (SPM), Raman amplifiers, and Raman scattering, along with ML techniques to upgrade the optical biosensors in detecting the SARS-CoV-2 virus efficiently, with a higher sensitivity [138]. This study presents the future opportunities of nonlinear optics processes in biosensing platforms.

## 3. Discussion of Future Perspectives of Optical Techniques

As refractive-index-based interferometric techniques play an important role in the detection of viruses, a non-invasive, label-free, optical coherence tomography (OCT) technique, which is based on Michelson interferometry, could be a potential diagnosis system for indirect detection of change in the refractive index of saliva in the oral tissue, as saliva has been proved to be one of the biomarkers of coronavirus [137]. In addition, nano-sensitive OCT (Ns-OCT) is an emerging depth-resolved novel technique, which is used to detect nanoscale levels of 30 nm without the use of labels/contrast agents [139]. This approach has been used for the detection of structural changes in corneal wound healing and can also be a valuable technique in the diagnosis of early stage cancer and the detection of viruses [140]. Another optical technique, label-free imaging of 75 nm adenovirus using submerged microsphere optical nanoscopy (SMON), has been reported elsewhere and uses the mechanism of frustrated total internal reflection using BaTiO_3_ microspheres [141]. Other microscopic techniques of super-resolution include structured illumination microscopy (SIM), photo-activation localization microscopy (PALM), and direct stochastic optical reconstruction microscopy (dSTORM) [142,143]. These methods overcome the optical diffraction limitation of 200 nm in standard optical microscopes and can be used as an alternative approach to electron and optical fluorescent microscopy for the detection of SARS-CoV-2 of less than 100 nm.

Several innovative testing methods are being researched currently by several research groups worldwide for rapid, non-invasive, and early detection of COVID-19. One of them is the laser-based saliva nasal swab test being developed by European photonics scientists [144]. The scientists have claimed to have developed a portable (25 × 15 × 25 cm) nano-interferometric biosensor instrument. In this method, the surface of the biosensor consists of bioreceptors, which are tuned to a particular antigen of the virus. As the light travels in the sensor, an evanescent field over a few nanometers gets generated. The direction of the light’s travel changes when the bioreceptor (antibody or the DNA strands) recognizes the antigen of the virus capsid. This patented technique is claimed to have the potential to detect coronavirus at the picomolar-to-attomolar range. Elsewhere, the detection of the whole virus in 30 min using the optical method has been the goal of the European-Union-supported CONVAT project, in which the viral content per mL in saliva samples was determined, followed by PCR testing [144]. In addition to saliva, viruses can also be detected from the condensate of exhaled breath [145,146]. One such example is the detection of influenza virus RNA in the breath exhale using an Exhalair device integrated with an optical particle counter at a generation rate ranging from less than 3.2 to 20 virus particles/min [147].

## 4. Conclusions

In addition to the diagnostic applications of nanoparticles in the optical detection of viruses, they also have a great potential in therapeutic applications against viruses because of their high surface-to-volume ratio, which allows them to interfere with and block viral entry into the cell [148,149]. It requires engineered nanomaterials to inactivate the viruses or inhibit viral binding to the surface receptor of the host cell. Carbon quantum dots (CQDs) of average diameter below 10 nm have been employed to study the antiviral properties and inhibition of viral activities related to human coronavirus HCoV-229E [150]. However, further in vivo experimental studies to validate its function on other coronaviruses are essential. A nanomaterial-based photodynamic therapy (PDT) has also been suggested to be an effective therapeutic method for viruses [151,152]. PDT is an approved technique used in cancer treatment [153]. It requires a photosensitizer, which when excited by a visible light reacts with dioxygen, forming reactive oxygen species (ROS) that can in turn react with biological molecules such as proteins, lipids, and nucleic acids, causing oxidation and finally leading to irreversible damage to the cells and tissues. Delivery of photosensitizers such as indocyanine green into the cells of the lungs and intra-tracheal and laser therapy or activation by an 810 nm laser source has been suggested to be a potential method against coronavirus [154].

This review paper provides an overview of various optical techniques, such as optical spectroscopic, nanomaterial-based, and interferometric optical biosensors, used for the detection of different types of viruses. More initiatives are required to transform these benchtop optical systems with extensive instrumentation constrained for laboratory use into miniaturized and portable optical setups reaching the market level. The importance of point-of-care diagnosis using lab-on-a-chip-based biosensors, as well as biosensors integrated with smartphones for real-time remote monitoring, has also been highlighted. The emergence of AI and its capability in handling big healthcare data in both the imaging and sensing fields for the diagnosis and screening of COVID-19 has been described. Discussion of future perspectives includes the potential of optical interferometric techniques in the non-invasive detection of viruses from other biological samples such as saliva and exhaled breath. Furthermore, the advantages of super-resolution optical microscopes and the use of nano-sensitive OCT for label-free detection of particles in the order of nanometers have been mentioned.

## Figures and Tables

**Table 1:** The history of viral pandemic diseases since 1918, their source of origin, and the number of deaths caused [1,2,3,4,5,6,7,8,9,10,11,12,13,14,15].

Year	Name of the Pandemic	Suspected Source of Origin	Number of Deaths
1918	Spanish Flu (H1N1 virus)	Pigs	20–100 million
1957	Asian Flu (H2N2 virus)	Pigs/Chickens/Ducks	0.7–1.5 million
1960	HIV (AIDS virus)	Chimpanzees	35 million
1968	Hongkong Flu (H3N2 virus)	Strain of H2N2 virus	1 million
1974	Small Pox	Variola virus	500 million
2002	SARS (coronavirus)	Bats/Civets	774
2009	Swine Flu (H1N1 virus)	Pigs	284 K
2012	MERS (coronavirus)	Bats/Civets	850
2014	Ebolavirus	Wild animals	11.3 K
2019	COVID-19 (SARS-CoV-2)	Uncertain	6,951,677 (to date)

**Table 3:** This table summarizes the list of nanomaterial-based optical biosensors, their corresponding target viruses, and detection limits.

Technique	Biomolecule	Nanoparticle	Target Virus	Detection Limit	Ref(s)
Fluorescence	Antibody	QD-MP & Zr NPs	Coronavirus	79.15 EID/50 µL	[80]
LSPR	Antibody	AuNP	HBV	0.01 IU/mL	[82]
SERS	DNA	AuNP	Influenza A/H1N1 virus	97 PFU/mL	[83]
DLS	DNA	AuNP	HIV	1.8 aM	[84]
FRET	Antibody	Graphene oxide	HIV	2 nM	[85]
Fluorometric	Antibody	AuNP	HBV	8.3 ng/mL	[86]
Fluorometric	Antibody	AuNP	H5N1	0.09 ng/mL	[87]
LSPR and PPT	Nucleic acid	AuNIs	SARS-CoV-2	0.22 pM	[88]

SERS—surface enhanced Raman scattering, LSPR—localized surface plasmon resonance, DLS—dynamic light scattering, FRET—fluorescence resonance energy transfer, PPT—plasmonic photothermal; AuNP—gold nanoparticles, QD-MP—quantum dot magneto particles, Zr NPs—zirconium nanoparticles, AuNIs—gold nano islands; HBV—hepatitis B virus, H1N1—influenza virus, HIV—human immunodeficiency virus, SARS-CoV-2—severe acute respiratory syndrome corona virus—2, IU/mL—international units per milliliter, PFU/mL—plaque-forming unit per milliliter, aM—attomolar, EID—egg infective dose, nM—nanomolar, pM—picomolar.

**Table 4:** List of optical interferometry-based biosensors and the corresponding target virus types and sensitivities.

Optical Interferometric Technique	Virus Type	Detection Limit	Ref(s)
Handheld portable Young interferometry	Herpes simplex virus 1	8.5 × 10^2^ to 8.5 × 10^6^ particles/mL	[98]
Surface plasmon Resonance	Vesicular stomatitis virus	10^6^ PFU/mL	[107]
MZI	Avian influenza virus	10^4^ to 10^7^ particles/HA unit	[108,109]
MZI	SARS-CoV-2	20 ng/mL	[110]
MZI	SARS-CoV-2	26.8 pM	[111]
Young interferometry	Herpes simplex virus 1	10^3^ virus particles/mL	[98,99]
Hartman interferometry	Influenza A virus	2 × 10^6^ PFU/mL	[99]
IRIS	Vesicular stomatitis virus	3.5 × 10^5^ PFU/mL	[112]
ARROW	Zaire Ebolavirus	0.2 PFU/mL	[113]
Fiber optic interferometer	SARS-CoV-2	1 μg/mL	[114]

MZI—Mach–Zehnder interferometer, IRIS—interferometric reflectance imaging sensor, ARROW—antiresonant reflecting optical waveguides, PFU/mL—plaque forming unit per milliliter.

## Data Availability

Not applicable.

## References

[1] WHO Coronavirus (COVID-19) Dashboard. https://covid19.who.int/.

[2] De Wit E., Van Doremalen N., Falzarano D., Munster V.J. (2016). SARS and MERS: Recent insights into emerging coronaviruses. Nat. Rev. Microbiol..

[3] Zhong N.S., Zheng B.J., Li Y.M., Poon L.L.M., Xie Z.H., Chan K.H., Li P.H., Tan S.Y., Chang Q., Xie J.P. (2003). Epidemiology and cause of severe acute respiratory syndrome (SARS) in Guangdong, People’s Republic of China, in February, 2003. Lancet.

[4] Zaki A.M., Van Boheemen S., Bestebroer T.M., Osterhaus A.D., Fouchier R.A. (2012). Isolation of a novel coronavirus from a man with pneumonia in Saudi Arabia. N. Engl. J. Med..

[5] Huang C., Wang Y., Li X., Ren L., Zhao J., Hu Y., Zhang L., Fan G., Xu J., Gu X. (2020). Clinical features of patients infected with 2019 novel coronavirus in Wuhan, China. Lancet.

[6] Bloom D.E., Cadarette D. (2019). Infectious disease threats in the twenty-first century: Strengthening the global response. Front. Immunol..

[7] Patterson K.D., Pyle G.F. (1991). The geography and mortality of the 1918 influenza pandemic. Bull. Hist. Med..

[8] Johnson N.P., Mueller J. (2002). Updating the accounts: Global mortality of the 1918–1920 “Spanish” influenza pandemic. Bull. Hist. Med..

[9] Saunders-Hastings P.R., Krewski D. (2016). Reviewing the history of pandemic influenza: Understanding patterns of emergence and transmission. Pathogens.

[10] Zhu T., Korber B.T., Nahmias A.J., Hooper E., Sharp P.M., Ho D.D. (1998). An African HIV-1 sequence from 1959 and implications for the origin of the epidemic. Nature.

[11] Barré-Sinoussi F., Chermann J.C., Rey F., Nugeyre M.T., Chamaret S., Gruest J., Dauguet C., Axler-Blin C., Vézinet-Brun F., Rouzioux C. (1983). Isolation of a T-lymphotropic retrovirus from a patient at risk for acquired immune deficiency syndrome (AIDS). Science.

[12] Weinraub B. (1974). Smallpox Grows in India; Worst Over, Officials Say. New York Times.

[13] Olsen S.J., Chang H.L., Cheung T.Y.Y., Tang A.F.Y., Fisk T.L., Ooi S.P.L., Kuo H.W., Jiang D.D.S., Chen K.T., Lando J. (2003). Transmission of the severe acute respiratory syndrome on aircraft. N. Engl. J. Med..

[14] Dawood F.S., Iuliano A.D., Reed C., Meltzer M.I., Shay D.K., Cheng P.Y., Bandaranayake D., Breiman R.F., Brooks W.A., Buchy P. (2012). Estimated global mortality associated with the first 12 months of 2009 pandemic influenza A H1N1 virus circulation: A modelling study. Lancet Infect. Dis..

[15] Liu Y.C., Kuo R.L., Shih S.R. (2020). COVID-19: The first documented coronavirus pandemic in history. Biomed. J..

[16] Naqvi A.A.T., Fatima K., Mohammad T., Fatima U., Singh I.K., Singh A., Atif S.M., Hariprasad G., Hasan G.M., Hassan M.I. (2002). Insights into SARS-CoV-2 genome, structure, evolution, pathogenesis and therapies: Structural genomics approach. Biochim. Biophys. Acta BBA-Mol. Basis Dis..

[17] Cui J., Li F., Shi Z.L. (2019). Origin and evolution of pathogenic coronaviruses. Nat. Rev. Microbiol..

[18] Pellett P.E., Mitra S., Holland T.C. (2014). Basics of virology. Handb. Clin. Neurol..

[19] Lau Y.L., Peiris J.M. (2005). Pathogenesis of severe acute respiratory syndrome. Curr. Opin. Immunol..

[20] Belosi F., Conte M., Gianelle V., Santachiara G., Contini D. (2021). On the concentration of SARS-CoV-2 in outdoor air and the interaction with pre-existing atmospheric particles. Environ. Res..

[21] Kevadiya B.D., Machhi J., Herskovitz J., Oleynikov M.D., Blomberg W.R., Bajwa N., Soni D., Das S., Hasan M., Patel M. (2021). Diagnostics for SARS-CoV-2 infections. Nat. Mater..

[22] Huang Y., Ding Z., Chen Q., Wu L., Guo L., Zhao C., Sha L., Sun H. (2021). Environmental virus detection associated with asymptomatic SARS-CoV-2-infected individuals with positive anal swabs. Sci. Total Environ..

[23] Xu R., Cui B., Duan X., Zhang P., Zhou X., Yuan Q. (2020). Saliva: Potential diagnostic value and transmission of 2019 nCoV. Int. J. Oral Sci..

[24] Pan M., Lednicky J.A., Wu C.Y. (2019). Collection, particle sizing and detection of airborne viruses. J. Appl. Microbiol..

[25] Ranjan P., Singhal A., Yadav S., Kumar N., Murali S., Sanghi S.K., Khan R. (2021). Rapid diagnosis of SARS-CoV-2 using potential point-of-care electrochemical immunosensor: Toward the future prospects. Int. Rev. Immunol..

[26] MacIntyre C.R., Chughtai A.A. (2020). A rapid systematic review of the efficacy of face masks and respirators against coronaviruses and other respiratory transmissible viruses for the community, healthcare workers and sick patients. Int. J. Nurs. Stud..

[27] Augustine R., Das S., Hasan A., Abdul Salam S., Augustine P., Dalvi Y.B., Varghese R., Primavera R., Yassine H.M., Thakor A.S. (2020). Rapid antibody-based COVID-19 mass surveillance: Relevance, challenges, and prospects in a 753 pandemic and post-pandemic world. J. Clin. Med..

[28] Teymouri M., Mollazadeh S., Mortazavi H., Ghale-Noie Z.N., Keyvani V., Aghababaei F., Hamblin M.R., Abbaszadeh-Goudarzi G., Pourghadamyari H., Hashemian S.M.R. (2021). Recent advances and challenges of RT-PCR tests for the diagnosis of COVID-19. Pathol. Res. Pract..

[29] Rajinikanth V., Dey N., Raj A.N.J., Hassanien A.E., Santosh K.C., Raja N. (2020). Harmony-search and otsu based system for coronavirus disease (COVID-19) detection using lung CT scan images. arXiv.

[30] Sreepadmanabh M., Sahu A.K., Chande A. (2020). COVID-19: Advances in diagnostic tools, treatment strategies, and vaccine development. J. Biosci..

[31] Han T., Cong H., Shen Y., Yu B. (2021). Recent advances in detection technologies for COVID-19. Talanta.

[32] Udugama B., Kadhiresan P., Kozlowski H.N., Malekjahani A., Osborne M., Li V.Y., Chen H., Mubareka S., Gubbay J.B., Chan W.C. (2020). Diagnosing COVID-19: The disease and tools for detection. ACS Nano.

[33] Maddali H., Miles C.E., Kohn J., O’Carroll D.M. (2021). Optical biosensors for virus detection: Prospects for SARS-CoV-2/COVID-19. ChemBioChem.

[34] Santos M.C., Morais C.L., Nascimento Y.M., Araujo J.M., Lima K.M. (2017). Spectroscopy with computational analysis in virological studies: A decade (2006–2016). TrAC Trends Anal. Chem..

[35] Chaudhary I., Jackson N., Denning D., O’Neill L., Byrne H.J. (2022). Contributions of vibrational spectroscopy to virology: A review. Clin. Spectrosc..

[36] Moor K., Ohtani K., Myrzakozha D., Zhanserkenova O., Andriana B.B., Sato H. (2014). Noninvasive and label-free determination of virus infected cells by Raman spectroscopy. J. Biomed. Opt..

[37] Santos M.C., Monteiro J.D., Araújo J.M., Lima K.M. (2020). Molecular fluorescence spectroscopy with multi-way analysis techniques detects spectral variations distinguishing uninfected serum versus dengue or chikungunya viral infected samples. Sci. Rep..

[38] Rodríguez-Casado A., Bartolomé J., Carreño V., Molina M., Carmona P. (2006). Structural characterization of the 5′ untranslated RNA of hepatitis C virus by vibrational spectroscopy. Biophys. Chem..

[39] Kim H., Hwang J., Kim J.H., Lee S., Kang M. Sensitive detection of multiple fluoresence probes based on surface-enhanced Raman scattering (sers) for mers-cov. Proceedings of the 2019 IEEE 14th International Conference on Nano/Micro Engineered and 776 Molecular Systems (NEMS).

[40] Sakudo A., Suganuma Y., Sakima R., Ikuta K. (2012). Diagnosis of HIV-1 infection by near-infrared spectroscopy: Analysis using molecular clones of various HIV-1 subtypes. Clin. Chim. Acta.

[41] Amathieu R., Triba M.N., Goossens C., Bouchemal N., Nahon P., Savarin P., Le Moyec L. (2016). Nuclear magnetic resonance based metabolomics and liver diseases: Recent advances and future clinical applications. World J. Gastroenterol..

[42] Slupsky C.M. (2011). Nuclear magnetic resonance-based analysis of urine for the rapid etiological diagnosis of pneumonia. Expert Opin. Med. Diagn..

[43] Lambert P.J., Whitman A.G., Dyson O.F., Akula S.M. (2006). Raman spectroscopy: The gateway into tomorrow’s virology. Virol. J..

[44] Salman A., Shufan E., Zeiri L., Huleihel M. (2014). Characterization and detection of Vero cells infected with Herpes Simplex Virus type 1 using Raman spectroscopy and advanced statistical methods. Methods.

[45] Kelly J.G., Trevisan J., Scott A.D., Carmichael P.L., Pollock H.M., Martin-Hirsch P.L., Martin F.L. (2011). Biospectroscopy to metabolically profile biomolecular structure: A multistage approach linking computational analysis with biomarkers. J. Proteome Res..

[46] Granger J.H., Schlotter N.E., Crawford A.C., Porter M.D. (2016). Prospects for point-of-care pathogen diagnostics using surface-enhanced Raman scattering (SERS). Chem. Soc. Rev..

[47] Auner G.W., Shanley C., Brusatori M., Twomey T., Sant D. (2019). Seraph Biosciences Inc.; Wayne State University. Hand-Held Micro-Raman Based Detection Instrument and Method of Detection. U.S. Patent.

[48] Saade J., Pacheco M.T.T., Rodrigues M.R., Silveira L. (2008). Identification of hepatitis C in human blood serum by near-infrared Raman spectroscopy. Spectroscopy.

[49] Santos M.C., Nascimento Y.M., Araújo J.M., Lima K.M. (2017). ATR-FTIR spectroscopy coupled with multivariate analysis techniques for the identification of DENV-3 in different concentrations in blood and serum: A new approach. Rsc. Adv..

[50] Santos M.C., Morais C.L., Lima K.M. (2020). ATR-FTIR spectroscopy for virus identification: A powerful alternative. Biomed. Spectrosc. Imaging.

[51] Kitane D.L., Loukman S., Marchoudi N., Fernandez-Galiana A., El Ansari F.Z., Jouali F., Badir J., Gala J.L., Bertsimas D., Azami N. (2021). A simple and fast spectroscopy-based technique for COVID-19 diagnosis. Sci. Rep..

[52] Desai S., Mishra S.V., Joshi A., Sarkar D., Hole A., Mishra R., Dutt S., Chilakapati M.K., Gupta S., Dutt A. (2020). Raman spectroscopy-based detection of RNA viruses in saliva: A preliminary report. J. Biophotonics.

[53] Barauna V.G., Singh M.N., Barbosa L.L., Marcarini W.D., Vassallo P.F., Mill J.G., Ribeiro-Rodrigues R., Campos L.C., Warnke P.H., Martin F.L. (2021). Ultrarapid on-site detection of SARS-CoV-2 infection using simple ATR-FTIR spectroscopy and an analysis algorithm: High sensitivity and specificity. Anal. Chem..

[54] Howes P.D., Chandrawati R., Stevens M.M. (2014). Colloidal nanoparticles as advanced biological sensors. Science.

[55] Baptista P., Pereira E., Eaton P., Doria G., Miranda A., Gomes I., Quaresma P., Franco R. (2008). Gold nanoparticles for the development of clinical diagnosis methods. Anal. Bioanal. Chem..

[56] Neng J., Harpster M.H., Wilson W.C., Johnson P.A. (2013). Surface-enhanced Raman scattering (SERS) detection of multiple viral antigens using magnetic capture of SERS-active nanoparticles. Biosens. Bioelectron..

[57] Cao Y.C., Jin R., Mirkin C.A. (2002). Nanoparticles with Raman spectroscopic fingerprints for DNA and RNA detection. Science.

[58] Griffin J., Singh A.K., Senapati D., Rhodes P., Mitchell K., Robinson B., Yu E., Ray P.C. (2009). Size-and distance-dependent nanoparticle surface-energy transfer (NSET) method for selective sensing of hepatitis C virus RNA. Chem. A Eur. J..

[59] Lu X., Dong X., Zhang K., Han X., Fang X., Zhang Y. (2013). A gold nanorods-based fluorescent biosensor for the detection of hepatitis B virus DNA based on fluorescence resonance energy transfer. Analyst.

[60] Nasrin F., Chowdhury A.D., Takemura K., Lee J., Adegoke O., Deo V.K., Abe F., Suzuki T., Park E.Y. (2018). Single-step detection of norovirus tuning localized surface plasmon resonance-induced optical signal between gold nanoparticles and quantum dots. Biosens. Bioelectron..

[61] Chang Y.F., Wang S.F., Huang J.C., Su L.C., Yao L., Li Y.C., Wu S.C., Chen Y.M.A., Hsieh J.P., Chou C. (2010). Detection of swine-origin influenza A (H1N1) viruses using a localized surface plasmon coupled fluorescence fiber-optic biosensor. Biosens. Bioelectron..

[62] Ganbold E.O., Kang T., Lee K., Lee S.Y., Joo S.W. (2012). Aggregation effects of gold nanoparticles for single-base mismatch detection in influenza A (H1N1) DNA sequences using fluorescence and Raman measurements. Colloids Surf. B Biointerfaces.

[63] Draz M.S., Fang B.A., Li L., Chen Z., Wang Y., Xu Y., Yang J., Killeen K., Chen F.F. (2012). Hybrid nanocluster plasmonic resonator for immunological detection of hepatitis B virus. ACS Nano.

[64] Nasrin F., Chowdhury A.D., Takemura K., Kozaki I., Honda H., Adegoke O., Park E.Y. (2020). Fluorometric virus detection platform using quantum dots-gold nanocomposites optimizing the linker length variation. Anal. Chim. Acta.

[65] Draz M.S., Shafiee H. (2018). Applications of gold nanoparticles in virus detection. Theranostics.

[66] Campbell C.T., Kim G. (2007). SPR microscopy and its applications to high-throughput analyses of biomolecular binding events and their kinetics. Biomaterials.

[67] Liedberg B., Nylander C., Lunström I. (1983). Surface plasmon resonance for gas detection and biosensing. Sens. Actuators.

[68] Margheri G., D’Agostino R., Becucci L., Guidelli R., Tiribilli B., Del Rosso M. (2012). Surface plasmon resonance as detection tool for lipids lateral mobility in biomimetic membranes. Biomed. Opt. Express.

[69] Schmidt A.G., Lee K., Yang P.L., Harrison S.C. (2012). Small-molecule inhibitors of dengue-virus entry. PLoS Pathog..

[70] Watanabe K. (2011). Model for measurement of water layer thickness under lipid bilayers by surface plasmon resonance. Biomed. Opt. Express.

[71] Jahanshahi P., Sekaran S.D., Adikan F.R.M. (2015). Optical and analytical investigations on dengue virus rapid diagnostic test for IgM antibody detection. Med. Biol. Eng. Comput..

[72] Li Y., Lu C., Zhou S., Fauconnier M.L., Gao F., Fan B., Lin J., Wang F., Zheng J. (2020). Sensitive and simultaneous detection of different pathogens by surface-enhanced Raman scattering based on aptamer and Raman reporter co-mediated gold tags. Sens. Actuators B Chem..

[73] Ngo H.T., Wang H.-N., Fales A.M., Nicholson B.P., Woods C.W., VoDinh T. (2014). DNA bioassay-on-chip using SERS detection for dengue diagnosis. Analyst.

[74] Eivazzadeh-Keihan R., Pashazadeh-Panahi P., Mahmoudi T., Chenab K.K., Baradaran B., Hashemzaei M., Radinekiyan F., Mokhtarzadeh A., Maleki A. (2019). Dengue virus: A review on advances in detection and trends–from conventional methods to novel biosensors. Microchim. Acta.

[75] Dardir K., Wang H., Martin B.E., Atzampou M., Brooke C.B., Fabris L. (2020). SERS nanoprobe for intracellular monitoring of viral mutations. J. Phys. Chem. C.

[76] Lim J.Y., Nam J.S., Yang S.E., Shin H., Jang Y.H., Bae G.U., Kang T., Lim K.I., Choi Y. (2015). Identification of newly emerging influenza viruses by surface-enhanced Raman spectroscopy. Anal. Chem..

[77] Sánchez-Purrà M., Carré-Camps M., de Puig H., Bosch I., Gehrke L., Hamad-Schifferli K. (2017). Surface-enhanced Raman spectroscopy-based sandwich immunoassays for multiplexed detection of Zika and Dengue viral biomarkers. ACS Infect. Dis..

[78] Yadav S., Sadique M.A., Ranjan P., Kumar N., Singhal A., Srivastava A.K., Khan R. (2021). SERS based lateral flow immunoassay for point-of-care detection of SARS-CoV-2 in clinical samples. ACS Appl. Bio Mater..

[79] Zhang D., Zhang X., Ma R., Deng S., Wang X., Wang X., Zhang X., Huang X., Liu Y., Li G. (2021). Ultra-fast and onsite interrogation of Severe Acute Respiratory Syndrome Coronavirus 2 (SARS-CoV-2) in waters via surface enhanced Raman scattering (SERS). Water Res..

[80] Ahmed S.R., Kang S.W., Oh S., Lee J., Neethirajan S. (2018). Chiral zirconium quantum dots: A new class of nanocrystals for optical detection of coronavirus. Heliyon.

[81] Xie B.P., Qiu G.H., Hu P.P., Liang Z., Liang Y.M., Sun B., Bai L.P., Jiang Z.H., Chen J.X. (2018). Simultaneous detection of Dengue and Zika virus RNA sequences with a three-dimensional Cu-based zwitterionic metal–organic framework, comparison of single and synchronous fluorescence analysis. Sens. Actuators B Chem..

[82] Wang X., Li Y., Wang H., Fu Q., Peng J., Wang Y., Du J., Zhou Y., Zhan L. (2010). Gold nanorod-based localized surface plasmon resonance biosensor for sensitive detection of hepatitis B virus in buffer, blood serum and plasma. Biosens. Bioelectron..

[83] Chen H., Park S.G., Choi N., Moon J.I., Dang H., Das A., Lee S., Kim D.G., Chen L., Choo J. (2020). SERS imaging-based aptasensor for ultrasensitive and reproducible detection of influenza virus A. Biosens. Bioelectron..

[84] Zou L., Ling L. (2018). Ultrasensitive detection of HIV DNA with polymerase chain reaction–dynamic light scattering. Anal. Chem..

[85] Wu Y.M., Cen Y., Huang L.J., Yu R.Q., Chu X. (2014). Upconversion fluorescence resonance energy transfer biosensor for sensitive detection of human immunodeficiency virus antibodies in human serum. Chem. Commun..

[86] Zeng Q., Zhang Y., Liu X., Tu L., Kong X., Zhang H. (2012). Multiple homogeneous immunoassays based on a quantum dots–gold nanorods FRET nanoplatform. Chem. Commun..

[87] Li X., Lu D., Sheng Z., Chen K., Guo X., Jin M., Han H. (2012). A fast and sensitive immunoassay of avian influenza virus based on label-free quantum dot probe and lateral flow test strip. Talanta.

[88] Qiu G., Gai Z., Tao Y., Schmitt J., Kullak-Ublick G.A., Wang J. (2020). Dual-functional plasmonic photothermal biosensors for highly accurate severe acute respiratory syndrome coronavirus 2 detection. ACS Nano.

[89] Song M., Yang M., Hao J. (2021). Pathogenic virus detection by optical nanobiosensors. Cell Rep. Phys. Sci..

[90] Fan X., White I.M., Shopova S.I., Zhu H., Suter J.D., Sun Y. (2008). Sensitive optical biosensors for unlabeled targets: A review. Anal. Chim. Acta.

[91] John P., Vasa N.J., Unni S.N., Rao S.R. (2017). Glucose sensing in oral mucosa simulating phantom using differential absorption based frequency domain low-coherence interferometry. Appl. Opt..

[92] John P., Vasa N.J., Sujatha N. (2019). Glucose sensing in the anterior chamber of the human eye model using supercontinuum source based dual wavelength low coherence interferometry. Sens. Bio-Sens. Res..

[93] Hamed A.M. (2016). Image processing of corona virus using interferometry. Opt. Photonics J..

[94] Goddard J.M., Mandal S., Nugen S.R., Baeumner A.J., Erickson D. (2010). Biopatterning for label-free detection. Colloids Surf. B Biointerfaces.

[95] Huang M.C., Mateus C.F., Foley J.E., Beatty R., Cunningham B.T., Chang-Hasnain C.J. (2008). VCSEL optoelectronic biosensor for detection of infectious diseases. IEEE Photonics Technol. Lett..

[96] Pang Y., Song H., Cheng W. (2016). Using optical trap to measure the refractive index of a single animal virus in culture fluid with high precision. Biomed. Opt. Express.

[97] Xu J., Suarez D., Gottfried D.S. (2007). Detection of avian influenza virus using an interferometric biosensor. Anal. Bioanal. Chem..

[98] Ymeti A., Greve J., Lambeck P.V., Wink T., van Hövell S.W., Beumer T.A., Wijn R.R., Heideman R.G., Subramaniam V., Kanger J.S. (2007). Fast, ultrasensitive virus detection using a young interferometer sensor. Nano Lett..

[99] Ymeti A., Subramaniam V., Beumer T.A., Kanger J.S. (2007). An ultrasensitive young interferometer handheld sensor for rapid virus detection. Expert Rev. Med. Devices.

[100] Mulder H.K., Ymeti A., Subramaniam V., Kanger J.S. (2012). Size-selective detection in integrated optical interferometric biosensors. Opt. Express.

[101] Di Fabrizio E., Schlücker S., Wenger J., Regmi R., Rigneault H., Calafiore G., West M., Cabrini S., Fleischer M., Van Hulst N.F. (2016). Roadmap on biosensing and photonics with advanced nano-optical methods. J. Opt..

[102] Avci O., Lortlar Ü.N., Yalçın Özkumur A., Ünlü M.S. (2015). Interferometric reflectance imaging sensor (IRIS)—A platform technology for multiplexed diagnostics and digital detection. Sensors.

[103] Schneider B.H., Edwards J.G., Hartman N.F. (1997). Hartman interferometer: Versatile integrated optic sensor for label-free, real-time quantification of nucleic acids, proteins, and pathogens. Clin. Chem..

[104] Fan X., White I.M., Zhu H., Suter J.D., Oveys H. (2007). Overview of novel integrated optical ring resonator bio/chemical sensors. Laser Resonators and Beam Control IX.

[105] White I.M., Oveys H., Fan X., Smith T.L., Zhang J. (2006). Integrated multiplexed biosensors based on liquid core optical ring resonators and antiresonant reflecting optical waveguides. Appl. Phys. Lett..

[106] Zhu H., White I.M., Suter J.D., Zourob M., Fan X. (2008). Opto-fluidic micro-ring resonator for sensitive label-free viral detection. Analyst.

[107] Yanik A.A., Huang M., Kamohara O., Artar A., Geisbert T.W., Connor J.H., Altug H. (2010). An optofluidic nanoplasmonic biosensor for direct detection of live viruses from biological media. Nano Lett..

[108] Wagner E.K., Hewlett M.J., Bloom D.C., Camerini D. (1999). Basic Virology.

[109] Desselberger U. (1975). Relation of virus particle counts to the hemagglutinating activity of influenza virus suspensions measured by the HA pattern test and by use of the photometric HCU method. Arch. Virol..

[110] Angelopoulou M., Makarona E., Salapatas A., Misiakos K., Synolaki E., Ioannidis A., Chatzipanagiotou S., Ritvos M.A., Pasternack A., Ritvos O. (2022). Directly immersible silicon photonic probes: Application to rapid SARS-CoV-2 serological testing. Biosens. Bioelectron..

[111] Tan Q., Wu S., Liu Z., Wu X., Forsberg E., He S. (2022). High sensitivity detection of SARS-CoV-2 by an optofluidic hollow eccentric core fiber. Biomed. Opt. Express.

[112] Lopez C.A., Daaboul G.G., Vedula R.S., Özkumur E., Bergstein D.A., Geisbert T.W., Fawcett H.E., Goldberg B.B., Connor J.H., Ünlü M.S. (2011). Label-free multiplexed virus detection using spectral reflectance imaging. Biosens. Bioelectron..

[113] Cai H., Parks J.W., Wall T.A., Stott M.A., Stambaugh A., Alfson K., Griffiths A., Mathies R.A., Carrion R., Patterson J.L. (2015). Optofluidic analysis system for amplification-free, direct detection of Ebola infection. Sci. Rep..

[114] Szczerska M., Wityk P., Listewnik P. (2022). The SARS-CoV-2 specific IgG antibodies biophotonic sensor. J. Biophotonics.

[115] Pires N.M.M., Dong T., Hanke U., Hoivik N. (2014). Recent developments in optical detection technologies in lab-on-a-chip devices for biosensing applications. Sensors.

[116] Zhu H., Fohlerová Z., Pekárek J., Basova E., Neužil P. (2020). Recent advances in lab-on-a-chip technologies for viral diagnosis. Biosens. Bioelectron..

[117] Ahrberg C.D., Manz A., Neuzil P. (2016). Palm-sized device for point-of-care Ebola detection. Anal. Chem..

[118] Li Z., Zhao J., Wu X., Zhu C., Liu Y., Wang A., Deng G., Zhu L. (2019). A rapid microfluidic platform with real-time fluorescence detection system for molecular diagnosis. Biotechnol. Biotechnol. Equip..

[119] Bedin F., Boulet L., Voilin E., Theillet G., Rubens A., Rozand C. (2017). Paper-based point-of-care testing for cost-effective diagnosis of acute flavivirus infections. J. Med. Virol..

[120] Quesada-González D., Merkoçi A. (2018). Nanomaterial-based devices for point-of-care diagnostic applications. Chem. Soc. Rev..

[121] Dincer C., Bruch R., Costa-Rama E., Fernández-Abedul M.T., Merkoçi A., Manz A., Urban G.A., Güder F. (2019). Disposable sensors in diagnostics, food, and environmental monitoring. Adv. Mater..

[122] Cho S., Islas-Robles A., Nicolini A.M., Monks T.J., Yoon J.Y. (2016). In situ, dual-mode monitoring of organ-on-a-chip with smartphone-based fluorescence microscope. Biosens. Bioelectron..

[123] Giavazzi F., Salina M., Ceccarello E., Ilacqua A., Damin F., Sola L., Chiari M., Chini B., Cerbino R., Bellini T. (2014). A fast and simple label-free immunoassay based on a smartphone. Biosens. Bioelectron..

[124] Gallegos D., Long K.D., Yu H., Clark P.P., Lin Y., George S., Nath P., Cunningham B.T. (2013). Label-free biodetection using a smartphone. Lab Chip.

[125] Kawasaki D., Yamada H., Sueyoshi K., Hisamoto H., Endo T. (2022). Imprinted photonic crystal-film-based smartphone-compatible label-free optical sensor for SARS-CoV-2 testing. Biosensors.

[126] Geng Z., Zhang X., Fan Z., Lv X., Su Y., Chen H. (2017). Recent progress in optical biosensors based on smartphone platforms. Sensors.

[127] Roblyer D. (2020). Perspective on the increasing role of optical wearables and remote patient monitoring in the COVID-19 era and beyond. J. Biomed. Opt..

[128] El Asnaoui K., Chawki Y. (2021). Using X-ray images and deep learning for automated detection of coronavirus disease. J. Biomol. Struct. Dyn..

[129] Hemdan E.E.D., Shouman M.A., Karar M.E. (2020). Covidx-net: A framework of deep learning classifiers to diagnose COVID-19 in X-ray images. arXiv.

[130] Shi F., Wang J., Shi J., Wu Z., Wang Q., Tang Z., He K., Shi Y., Shen D. (2020). Review of artificial intelligence techniques in imaging data acquisition, segmentation, and diagnosis for COVID-19. IEEE Rev. Biomed. Eng..

[131] Xu X., Jiang X., Ma C., Du P., Li X., Lv S., Yu L., Ni Q., Chen Y., Su J. (2020). A deep learning system to screen novel coronavirus disease 2019 pneumonia. Engineering.

[132] Jamshidi M., Lalbakhsh A., Talla J., Peroutka Z., Hadjilooei F., Lalbakhsh P., Jamshidi M., La Spada L., Mirmozafari M., Dehghani M. (2020). Artificial intelligence and COVID-19: Deep learning approaches for diagnosis and treatment. IEEE Access.

[133] O’Connor T., Javidi B. (2022). COVID-19 screening with digital holographic microscopy using intra-patient probability functions of spatio-temporal bio-optical attributes. Biomed. Opt. Express.

[134] Shamout F.E., Shen Y., Wu N., Kaku A., Park J., Makino T., Jastrzębski S., Witowski J., Wang D., Zhang B. (2021). An artificial intelligence system for predicting the deterioration of COVID-19 patients in the emergency department. NPJ Digit. Med..

[135] Barnawi A., Chhikara P., Tekchandani R., Kumar N., Alzahrani B. (2021). Artificial intelligence-enabled Internet of Things-based system for COVID-19 screening using aerial thermal imaging. Future Gener. Comput. Syst..

[136] Kaushik A.K., Dhau J.S., Gohel H., Mishra Y.K., Kateb B., Kim N.Y., Goswami D.Y. (2020). Electrochemical SARS-CoV-2 sensing at point-of-care and artificial intelligence for intelligent COVID-19 management. ACS Appl. Bio Mater..

[137] Ember K., Daoust F., Mahfoud M., Dallaire F., Ahmad E.Z., Tran T., Plante A., Diop M.K., Nguyen T., St-Georges-Robillard A. (2022). Saliva-based detection of COVID-19 infection in a real-world setting using reagent-free Raman spectroscopy and machine learning. J. Biomed. Opt..

[138] To K.K.W., Tsang O.T.Y., Leung W.S., Tam A.R., Wu T.C., Lung D.C., Yip C.C.Y., Cai J.P., Chan J.M.C., Chik T.S.H. (2020). Temporal profiles of viral load in posterior oropharyngeal saliva samples and serum antibody responses during infection by SARS-CoV-2: An observational cohort study. Lancet Infect. Dis..

[139] Alexandrov S.A., Subhash H.M., Zam A., Leahy M. (2014). Nano-sensitive optical coherence tomography. Nanoscale.

[140] Lal C., Alexandrov S., Rani S., Zhou Y., Ritter T., Leahy M. (2020). Nanosensitive optical coherence tomography to assess wound healing within the cornea. Biomed. Opt. Express.

[141] Li L., Guo W., Yan Y., Lee S., Wang T. (2013). Label-free super-resolution imaging of adenoviruses by submerged microsphere optical nanoscopy. Light Sci. Appl..

[142] Chojnacki J., Eggeling C. (2018). Super-resolution fluorescence microscopy studies of human immunodeficiency virus. Retrovirology.

[143] Sydor A.M., Czymmek K.J., Puchner E.M., Mennella V. (2015). Super-resolution microscopy: From single molecules to supramolecular assemblies. Trends Cell Biol..

[144] Wallace J. New ‘Saliva Test’ to Instantly Detect Coronavirus with Lasers. Test and Measurement 2020, Laser Focus World. https://www.laserfocusworld.com/test-measurement/article/14173589/new-saliva-test-to-instantly-detect-coronavirus-via-interferometric-laser-technology.

[145] Ryan D.J., Toomey S., Madden S.F., Casey M., Breathnach O.S., Morris P.G., Grogan L., Branagan P., Costello R.W., De Barra E. (2021). Use of exhaled breath condensate (EBC) in the diagnosis of SARS-CoV-2 (COVID-19). Thorax.

[146] Sawano M., Takeshita K., Ohno H., Oka H. (2020). A short perspective on a COVID-19 clinical study: ‘Diagnosis of COVID-19 by RT-PCR using exhale breath condensate samples’. J. Breath Res..

[147] Fabian P., McDevitt J.J., DeHaan W.H., Fung R.O., Cowling B.J., Chan K.H., Leung G.M., Milton D.K. (2008). Influenza virus in human exhaled breath: An observational study. PLoS ONE.

[148] Carvalho L.F.d.C.e.S., Nogueira M.S. (2020). Optical techniques for fast screening-Towards prevention of the coronavirus COVID-19 outbreak. Photodiagnosis Photodyn. Ther..

[149] Rai M., Deshmukh S.D., Ingle A.P., Gupta I.R., Galdiero M., Galdiero S. (2016). Metal nanoparticles: The protective nanoshield against virus infection. Crit. Rev. Microbiol..

[150] Łoczechin A., Séron K., Barras A., Giovanelli E., Belouzard S., Chen Y.T., Metzler-Nolte N., Boukherroub R., Dubuisson J., Szunerits S. (2019). Functional carbon quantum dots as medical countermeasures to human coronavirus. ACS Appl. Mater. Interfaces.

[151] Wainwright M. (2003). Local treatment of viral disease using photodynamic therapy. Int. J. Antimicrob. Agents.

[152] Kharkwal G.B., Sharma S.K., Huang Y.Y., Dai T., Hamblin M.R. (2011). Photodynamic therapy for infections: Clinical applications. Lasers Surg. Med..

[153] Agostinis P., Berg K., Cengel K.A., Foster T.H., Girotti A.W., Gollnick S.O., Hahn S.M., Hamblin M.R., Juzeniene A., Kessel D. (2011). Photodynamic therapy of cancer: An update. CA Cancer J. Clin..

[154] Fekrazad R. (2020). Photobiomodulation and antiviral photodynamic therapy as a possible novel approach in COVID-19 management. Photobiomodulation Photomed. Laser Surg..

